# Ultrasonic-Assisted K^+^ Modification of Industrial Hemp Stalk Hydrothermal Biochar for Highly Effective Adsorption of Pb^2+^

**DOI:** 10.3390/ma18102348

**Published:** 2025-05-18

**Authors:** Le Liu, Wanjin Yu, Zheren Zhang, Qiyao Li, Chun Peng, Kaisheng Wu, Duoduo Liu, Sufang He, Nengsheng Liu, Xiang Li

**Affiliations:** 1 Faculty of Metallurgical and Energy Engineering, Kunming University of Science and Technology, Kunming 650093, China; 20222202184@stu.kust.edu.cn (L.L.); 20232202053@stu.kust.edu.cn (K.W.); 20232102041@stu.kust.edu.cn (D.L.); 2 State Key Laboratory of Fluorinated Greenhouse Gases Replacement and Control Treatment, Zhejiang Research Institute of Chemical Industry, Hangzhou 310023, China; yuwanjinhe@163.com; 3 Research Center for Analysis and Measurement, Kunming University of Science and Technology, Kunming 650093, China; zhangzr@idsse.ac.cn (Z.Z.); sufanghe@kust.edu.cn (S.H.); 4 Faculty of Materials Science and Engineering, Kunming University of Science and Technology, Kunming 650093, China; 20222230044@stu.kust.edu.cn (Q.L.); 20222130114@stu.kust.edu.cn (C.P.)

**Keywords:** industrial hemp stalk hydrothermal biochar, ultrasonic-assisted KOH activation, efficient adsorption, heavy metal

## Abstract

Biochar modification represents an effective approach for enhancing adsorption capacity. In the research, industrial hemp straw-derived biochar was synthesized through hydrothermal carbonization coupled with ultrasound-assisted KOH activation, demonstrating exceptional Pb^2+^ adsorption efficiency. The optimal HBS50-K0.5M exhibited excellent adsorption performance, achieving the maximum adsorption capacity of 345.8 mg/g within 2 h. The etching effect of KOH on the biochar surface increased the O-containing functional groups, which enhanced the adsorption of Pb^2+^. The adsorption kinetics revealed that the adsorption process of Pb^2+^ was aligned with the pseudo-second-order kinetics as well as the Langmuir model. The complexation, ion exchange, π-π interaction, as well as electrostatic interaction participated in the adsorption. This study demonstrates that ultrasound-assisted KOH-activated biochar has great potential for removing Pb^2+^ from wastewater.

## 1. Introduction

Lead is one of the most toxic and carcinogenic heavy metals. The discharge of lead without effective treatment poses a threat to the environment and human health [1]. Lead contamination not only damages soil and water quality but also enters living organisms through the food chain and water, causing serious damage to the central nervous system, kidneys, and reproductive system and increasing the risk of illness and even death [1]. In response, numerous conventional and innovative approaches have been employed to address wastewater treatment, like ion exchange processes, membrane filtration systems, chemical precipitation techniques, electrochemical treatment technologies, and reverse osmosis methods [2,3]. These techniques can meet discharge standards for lead-containing wastewater, but they have certain disadvantages, including insufficient treatment, costly equipment, high labor as well as monitoring costs, chemically intensive systems, the production of secondary contaminants (such as sludge and solid waste), as well as the requirement for additional treatment [4]. Sorption has been found to be an effective and convenient method. Among the many types of adsorbents, carbon materials are known for their excellent sorption stability with little or no secondary contamination [5,6]. In particular, carbon materials made from different biomasses (i.e., biochar) have received more attention because of their high adsorption capacity, easy synthesis, and low cost [7,8]. Thus, biochar has broad prospects in removing Pb^2+^.

Traditional biochar is synthesized from high-temperature pyrolysis in an oxygen-limited or quarantined condition, in which a series of reactions that degrades the organic macromolecules drastically diminishes the content of O, N, and H elements from the biomass for forming the porous carbon material is necessary [7]. However, the surface organic functional groups, which are the key heavy metal ions binding factor of biochar, would fall off dramatically in these violent reactions under high temperatures and undoubtedly weaken its sorption ability [8]. To compensate for this disadvantage, researchers have attempted to develop a hydrothermal carbonization method. The liquid hydrothermal medium will discharge a lot of H^+^ and OH^−^ in the hydrothermal environment with high pressure, comparatively low temperature, and an airtight chamber. This can catalyze the dehydration, deterioration, and breaking of organic macromolecules. The hydrothermal process offers a more moderate reaction condition compared to high-temperature pyrolysis for the formation of porous carbon material so that the organic functional groups in biomass can be reserved abundantly during the carbonization [7,8]. Therefore, hydrothermal biochar is considered a better adsorbent for heavy metals.

In a previous study, hydrochar was prepared from industrial hemp stalk waste cores. The 50% H_2_SO_4_-activated hydrochar (HBS50) exhibited a high adsorption capacity of 195.9 mg/L, demonstrating good performance [8]. However, further activation could significantly enhance its adsorption capability. According to the previous literature, the K^+^ ions have a significant activation effect on biochar. The K^+^ ions could infiltrate into the carbon lattice, react with organic matter, and release H_2_, CO, and CO_2_ gas, enlarging the pore structure and increasing the water permeation rate [9,10,11]. Meanwhile, K^+^ ions could corrode the surface of biochar and generate more active functional groups [12,13]. Moreover, the residual K atoms attached to biochar undergo ion exchange with Pb^2+^, which provides assistance in sorption [14].

However, traditional K^+^ modification is often accompanied by cumbersome procedures or strict temperature and gas shield requirements for pyrolysis. Generally, the biomass or prepared biochar is mixed with K^+^ solution, and the mixture is pyrolyzed in the range of 500 to 1000 °C under N_2_ protection after drying, which was incompatible with the currently advocated “energy saving as well as emission reduction” [15]. According to a report, ultrasonic-assisted modification has been established as a promising technique in materials engineering, where acoustic cavitation effects facilitate complete ion migration at reduced energy expenditure [16]. Therefore, we attempted to synthesize K^+^-modified biochar using ultrasonic oscillation for Pb^2+^ sorption. The effect of KOH concentrations on the physicochemical properties, Pb^2+^ sorption ability, as well as the mechanisms of biochars are studied systematically. The work provides a reference for the adsorption of heavy metals by modified biochar.

## 2. Experimental

### 2.1. Material

The industrial hemp stalk cores were gathered from the industrial hemp cultivation site in Chenggong, Yunnan University.

The chemicals used in this study, including HNO_3_, NaNO_3_, HCl, NaOH, KOH, H_2_SO_4_, CaCl_2_, and KCl were analytically pure and purchased from China National Pharmaceutical Chemical Reagent Company; Pb(NO_3_)_2_ was analytically pure and purchased from China Tianjin Fengchuan Chemical Reagent Technology Co., Tianjin, China. The water leveraged in the research was deionized water.

### 2.2. Prepared Material

The industrial hemp stalk cores were subjected to a drying process at 80 °C for a duration of 12 h. Subsequently, they were stripped, chopped into smaller pieces, and further pulverized to acquire industrial hemp stalk core (IHSC) powder with a diameter of less than 150 μm. Thereafter, 1.0 g of the obtained IHSC powder was blended with 25 mL of a 50% H_2_SO_4_ solution within a polytetrafluoroethylene (PTFE)-lined steel autoclave. The autoclave was then heated to 200 °C and maintained at this temperature for 12 h. Upon natural cooling, the resulting mixture was washed with deionized water and filtered repetitively until the washing solution achieved a clear and neutral state. Thereafter, the material obtained was subjected to a drying treatment and subsequently ground into fine particles, thus generating the hydrothermal biochar denoted as HBS50. Following that, 0.5 g of HBS50 was mixed separately with three different concentrations (0.25, 0.5, and 1 mol/L) of KOH solutions in amounts of 20 mL each. The suspensions were ultrasonicated for one hour and then stirred at 30 °C for 6 h. Finally, the mixture was washed and filtered until the filtrate became clear and neutral again. The KOH-modified hydrothermal biochar obtained after drying was named HBS50-KxM (x represents the concentration of KOH solution utilized).

### 2.3. Characterization

Microstructural and compositional characterization. Sample morphology was investigated using two scanning electron microscopy systems (Verios G4 UC, Thermo Scientific, Waltham, MA, USA, as well as Nova NanoSEM 450, FEI NanoPorts, Hillsboro, OR, USA). Elemental analysis was performed with LECO elemental analyzers (CS844 for carbon/sulfur and ONH836 for oxygen/nitrogen/hydrogen determination). Textural properties, including specific surface area (SBET), pore volume, as well as pore size distribution, were quantified through nitrogen physisorption measurements via a Micromeritics ASAP-2460 surface area analyzer. X-ray diffraction (XRD) patterns were attained via an XRD device (model: BRUKERD8, manufacturer: Bruker, country: USA) operating with Cu Kα radiation (wavelength, λ = 1.5406 Å) at 40 kV and 30 mA. The wide-angle XRD data were gathered across a 2θ range of 10°–90°, with a scanning rate of 8° per minute. FTIR spectroscopy was conducted on an FTIR spectrometer (model: Nicolet560 IR, manufacturer: Thermo Nicolet, Madison, WI, USA) at a resolution of 2 cm^−1^ covering a spectral range of 4000–400 cm^−1^. For X-ray photoelectron spectroscopy (XPS; model: ESCALAB250 Xi, manufacturer: Thermo Scientific, country: USA), the samples were pressed into pellets and loaded into the ultra-high vacuum to determine the elemental composition of carbon (C), oxygen (O), and lead (Pb) in the samples.

The zero-charge point (pHpzc) of biochar was assessed through the utilization of an adapted pH drift technique. Specifically, 0.04 g of biochar was introduced into conical flasks and combined with 50 mL of a 0.01 mol/L NaNO₃ solution, which had been adjusted to different initial pH levels (3, 4, 5, 6, 7, 8, and 9). The resulting mixtures were agitated at 25 °C for a duration of 24 h. The pHpzc was identified as the pH value where the plotted curve intersected the line, denoting equivalence between the initial and final pH values (pH_initial_ = pH_final_).

### 2.4. Sorption Experiment

Stock solutions with varying concentrations of Pb^2+^ (ranging from 20 to 500 mg/L) were formulated by dissolving Pb(NO_3_)_2_ as the dissolving agent in deionized water. Subsequently, the pH of these solutions was adjusted using 0.1 mol/L NaOH and 0.1 mol/L HNO₃.

Batch experiments examining Pb^2+^ sorption onto activated hydrothermal biochar were conducted by combining 25 mL of a Pb^2+^ solution (500 mg/L, pH = 5) with 0.02 g of biochar in a conical flask. The mixture was then agitated using a magnetic stirrer at 25 °C for a duration of 2 h.

Additional sorption trials were executed on HBS50-K0.5M to assess the influence of varying parameters, including pH level, adsorbent quantity, Pb^2+^ concentration, and interaction duration, on sorption efficacy. The adsorbent quantities evaluated were 0.01, 0.02, 0.03, and 0.04 g; pH levels tested were 2, 3, 4, 5, as well as 6; Pb^2+^ concentrations ranged from 20 to 500 mg/L in increments (20, 40, 80, 120, 160, 200, 300, 500 mg/L); and interaction times spanned 10 to 1440 min in specified intervals (10, 20, 40, 60, 120, 240, 480, 720, 1440 min).

Following the predetermined sorption duration, the suspensions were filtered, and an inductively coupled plasma optical emission spectrometer was leveraged to measure the Pb^2+^ concentrations in the solution both before and after sorption. The Pb^2+^ sorption capacity was computed through the application of Equations (1) and (2), as delineated below.

(1) $$q=\frac{\left(c_{o}-c_{t}\right)\times v}{m}$$

(2) $$\eta =\frac{c_{0}-c_{t}}{c_{0}}\times 100\%$$

where *q* (mg/g) is the sorption capacity of sorbent for Pb^2+^, *C_0_* (mg/L) is the initial concentration of Pb^2+^, *C_t_* (mg/L) is the concentration of Pb^2+^, *V* (L) is the volume of sorption solution, *m* (g) is the mass of biochar utilized. *η* (%) is the removal efficiency for Pb^2+^.

Based on the sorption experiment results for HBS50-K0.5M, sorption kinetics, as well as isotherm parameters, were determined and plotted via the pseudo-first-order (PF) and pseudo-second-order (PS) kinetic models, together with the Langmuir and Freundlich isotherm models. The relevant equations are detailed as follows (Equations (3)–(6)):

(3) $$\mathrm{PF}:\;In\left(q_{e}-q_{t}\right)=Inq_{e}-k_{1}\times t$$

(4) $$\mathrm{PS}:\;\frac{t}{q_{t}}=\frac{1}{k_{2}\times q_{e}^{2}}+\frac{t}{q_{e}}$$

where *q_e_* (mg/g) and *q_t_* (mg/g) represent the adsorption capacity at equilibrium and at time (min), while *k*_1_ and *k*_2_ (min^−1^) denote the rate constants for the PF as well as PS kinetic models. By plotting time (t) on the x-axis and either ln(*q_e_* − *q_t_*) or (t/*q_t_*) on the y-axis, a linear regression can be performed on the experimental data. The kinetic parameters are then determined from the slope as well as the intercept of the fitted line.

(5) $$\mathrm{Langmuir}\;\mathrm{isotherm}:\;q_{e}=\frac{q_{m}\times k_{l}\times c_{e}}{1+k_{l}\times c_{e}}$$

(6) $$\mathrm{Freundlich}\;\mathrm{isotherm}:\;q_{e}=k_{f}\times c_{e}^{1/n}$$

where *q_e_* (mg/g) denotes the equilibrium adsorption capacity, while *c_e_* (mg/L) represents the solute concentration in solution at equilibrium. The parameter *qₘ* (mg/g) corresponds to the theoretical maximum adsorption capacity. Additionally, *k_l_* is the Langmuir isotherm constant, *k_f_* is the Freundlich constant, and 1/n (dimensionless) characterizes the adsorption intensity.

## 3. Results and Discussion

### 3.1. Physicochemical Properties of KOH-Modified Industrial Hemp Stalk Core Hydrothermal Biochar

The makeup of diverse constituents within the industrial hemp stalk core (IHSC), HBS50, and hydrothermal biochar modified with ultrasonic-assisted KOH is displayed in Table 1. Compared to IHSC, the increasing C content and decreasing O content in HBS50 can be observed, which may be associated with degradation, pyrolysis, and dehydration reactions of organic macromolecules during hydrothermal processes. The O content in HBS50-KxM increased after ultrasonic treatment with KOH compared with HBS50, suggesting the increase of oxygen-containing groups on the biochar surface. Nevertheless, when the concentration of KOH solution was increased to 1 mol/L, the content of elemental oxygen decreased, which may be because of the damage of O-containing functional groups on the biochar surface by excessive KOH etching [10]. According to the previous literature, the surface of carbon materials was attacked and destroyed by K ions (relevant reaction as following Equations (7) to (11)), leading to an increase in active sites on the surface, as well as the generation of abundant functional groups.

6KOH + 2C → 2K + 3H_2_ + 2K_2_CO_3_ (7)

K_2_CO_3_ → K_2_O + CO_2_ (8)

CO_2_ + C → 2CO (9)

K_2_CO_3_ + 2C → 2K + 3CO (10)

C + K_2_O → 2K + CO (11)

**Figure 1:**
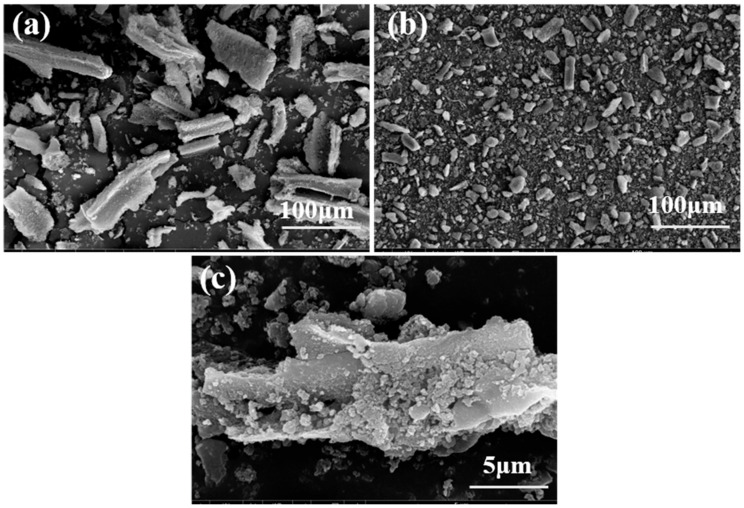
SEM images of HBS50 and HBS50−K0.5M: (**a**–**c**) HBS50−K0.5M.

The morphology of HBS50 and HBS50-K0.5M was characterized by SEM. From Figure 1a,b, it can be seen that the dispersion of the material was significantly improved after ultrasonic-assisted KOH activation. This change may be ascribed to the etching effect of KOH on the biochar, which disrupts the structure of the biochar and generates smaller biochar fragments [12,13]. Under high magnification observation, HBS50-K0.5M has a rough morphology and stereoscopic properties (Figure 1c). This is because the K ions undergo a series of reactions during the modification process that erode the carbon chain structure, enlarge the carbon lattice, and release gases, thus forming a surface with abundant organic functional groups, which is favorable for the adsorption of pb^2+^ [12,13].

SEM characterization revealed distinct morphological differences between HBS50 and KOH-modified HBS50-K0.5M. Figure 1 clearly demonstrates the enhanced dispersion of HBS50-K0.5M, resulting from KOH-induced etching of the carbon matrix. The specific surface area and pore structure of biochar are examined utilizing the Brunauer–Emmett–Teller (BET) technique and compiled in Table 1. As the KOH concentration increased, the specific surface area, total pore volume, and average pore diameter displayed visible changes. The average pore size of HBS50-KxM has increased from 8.95 nm to 18.65–19.15 nm, which might be related to a series of reactions—between KOH and biochar, releasing CO, CO_2_, and other gases. These gases overflowed from the suspension, expanding the pore size of the biochar.

**Figure 2:**
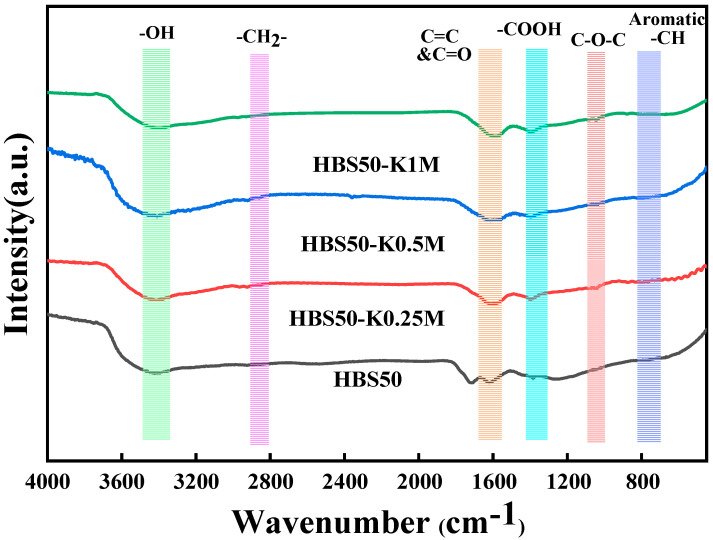
FTIR spectra of HBS50 and KOH−modified industrial hemp stalk core hydrothermal biochar.

FTIR results of biochars are displayed in Figure 2, which illustrates the functional groups of HBS50, HBS50-K0.25M, HBS50-K0.5M, and HBS50-K1M. The -OH bonds between the hydroxyl group and the H_2_O molecule were stretched, resulting in broad and strong bands at 3450 cm^−1^ [17]. The -CH_2_ bonds were associated with the peaks at 2850 cm^−1^ [18]. The peaks near 1680 and 1590 cm^−1^ were due to the stretching vibrations of C=O and C=C [17,18]. The stretching vibration of -COO generates peaks near 1390 and 1250 cm^−1^, while the C-O-C bond appeared near the 1060 cm^−1^ peak [19]. Aromatic C-H is attributed to peaks at 800–600 cm^−1^ [10]. Notably, the adsorption peak enhancement of C-O-C and -COOH was observed in HBS50-KxM, indicating an increase in O-containing groups, which is in agreement with the results of the elemental analysis.

The point of zero charge (pH_pzc_) corresponds to the pH level at which the net surface charge reaches neutrality, serving as a key indicator for assessing the surface charge properties of biochar. After activation in KOH solutions of different concentrations (0.25, 0.5, 1 mol/L), the pH_pzc_ of HBS50 exhibited a slight increase from 3.35 to a range between 4.33 and 4.49, as indicated in Table 1. In previous literature, the presence of residual alkali metal elements in biochar has been found to impact the pH_pzc_ of biochar [20]. The K^+^ ions in solution may be adsorbed through complexation reactions with -COOH or -OH to produce -COOK and -OK groups (as Equations (12) and (13)). Thereby, the number of surface groups that could provide H^+^ for deprotonation was reduced. Besides that, some K^+^ ions may also attach to biochar surfaces or pores in the form of electrostatic sorption [10]. For these reasons, the pH_pzc_ of KOH-modified biochar increased.

-COOH + K^+^ → -COOK + H^+^ (12)

-OH + K^+^ → -OK + H^+^ (13)

In summary, the activation of KOH has a significant impact on the physical and chemical properties of hemp core biochar synthesized by the hydrothermal carbonization method and thus affects the sorption characteristics of hydrothermal biochar for Pb^2+^.

### 3.2. Adsorption Ability and Performance of Ultrasonic-Assisted K^+^-Modified Hydrothermal Biochar in Industrial Hemp Stalk Core

#### 3.2.1. Result of Batch Adsorbent Experiment

**Figure 3:**
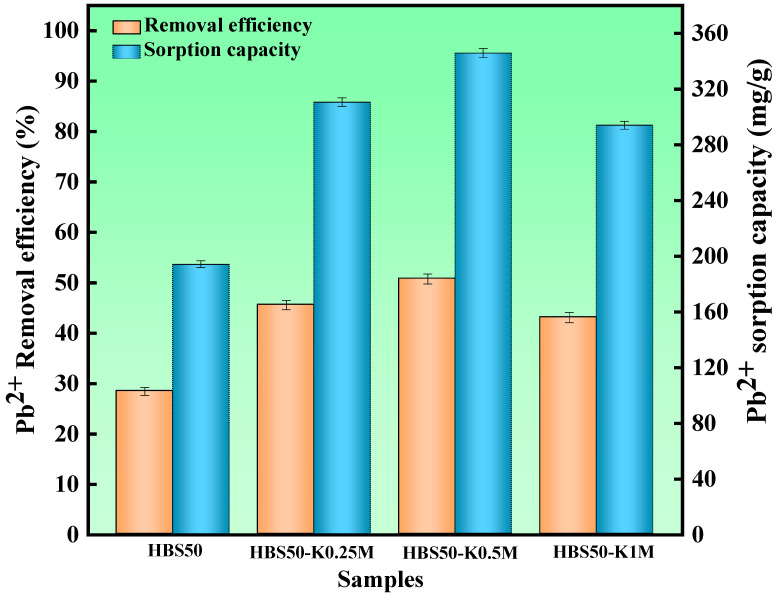
The Pb^2+^ removal experiments for HBS50 and KOH-ultrasonically modified carbon materials were conducted under the following conditions: adsorbent dosage = 20 mg, initial pH = 5, temperature = 25 °C, time = 120 min, solution volume = 25 mL.

**Figure 4:**
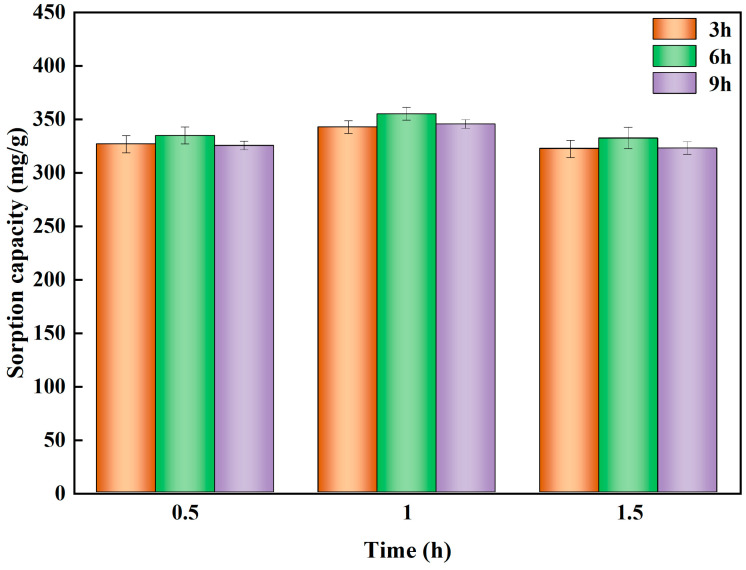
Experimental conditions: adsorbent dosage = 20 mg, initial pH = 5, temperature = 25 °C, time = 120 min, solution volume = 25 mL.

To determine the effect of different KOH solution concentrations on the sorbent performance in hydrothermal biochar. HBS50, HBS50-K0.25M, HBS50-K0.5M, and HBS50-K1M were used for sorbent experiments. As illustrated in Figure 3, the results of Pb^2+^ sorbent experiments on unmodified hydrothermal biochar and ultrasonic treatment by different KOH concentrations were presented. The Pb^2+^ sorption capacity of hydrothermal biochar was significantly improved through KOH ultrasonic treatment. Among them, the hydrothermal biochar (HBS50-K0.5M) treated by ultrasonic methods with 0.5 mol/L KOH solution had the best Pb^2+^ sorption capacity and removal rate (345.8 mg/g and 50.76%). Meanwhile, a preliminary investigation was conducted to determine the preparation conditions (sonication time and stirring time) of HBS50-K0.5M (Figure 4). HBS50-K0.5M exhibited the highest adsorption capacity by sonicating for 1 h and stirring for 6 h. Increasing ultrasound and stir time caused a slight decrease, which may be related to the collapse of pores. The increase in sorbent capacity was attributed to the corrosion of the hydrothermal biochar surface during KOH ultrasonic treatment, leading to the formation of abundant functional groups, thus improving its sorbent capacity [12]. It was noteworthy that when the KOH concentration increased to 1 M, the sorbent capacity and efficiency of hydrothermal biochar for Pb^2+^ were reduced. This was primarily because of the excessively high KOH concentration due to over-activation, causing the collapse of the carbon structure, destruction of carbon tissue, and loss of functional groups [21].

#### 3.2.2. Result of Further Adsorbent Experiments

To evaluate the influence of sorbent dosage, Pb^2+^ concentration, initial pH, as well as reaction time on the adsorption performance, systematic experiments were performed using HBS50-K0.5M.

**Figure 5:**
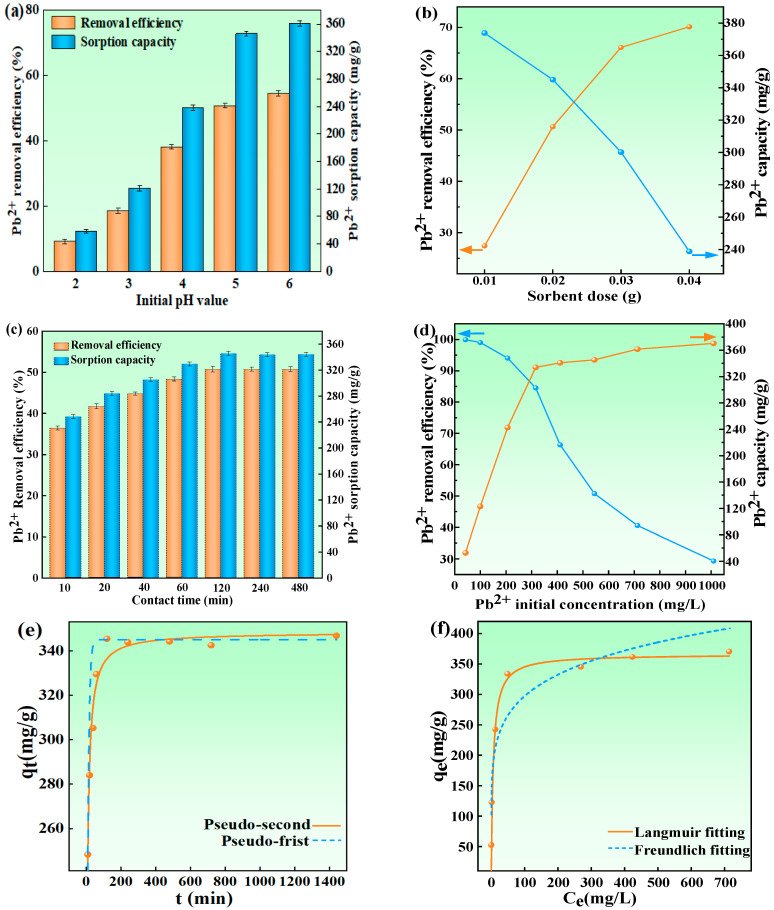
Impact of experimental variables on HBS50-K0.5M’s ability to remove Pb^2+^. Initial pH (**a**), dose of the adsorbent (**b**), reaction time (**c**,**d**), and initial Pb^2+^ concentration (**e**,**f**). Pb^2+^ concentrations of 0.02, 0.04, 0.08, 0.12, 0.16, 0.2, 0.3, and 0.5 g/L; reaction times of 10, 20, 40, 60, 120, 240, 480, 720, and 1440 min; sorbent quantities of 10, 20, 30, and 40 mg; beginning pH of 2, 3, 4, 5, and 6 were all employed in the experiment.

The pH affects the surface charge of sorbents, which affects the adsorption properties. According to previous research results, when pH > 6, Pb exists mainly as Pb(OH)_2_ precipitation. Therefore, the initial pH of Pb^2+^ solution was adjusted, ranging from 2 to 6 [22]. As displayed in Figure 5a, at the pH value of 2, a Pb^2+^ removal rate of 10.99% and sorbent capacity of 59.9 mg/g were observed. Subsequently, the removal rate and sorbent capacity increased with the initial pH of the solution. They increased to 50.76% and 354.8 mg/g when the pH was increased to 5, eventually reaching a maximum at a pH of 6 (54.43% and 361.3 mg/g). It was certain that higher pH values favor the sorption of Pb^2+^ because, in high pH environments, the -COOH, as well as -OH groups, on the surface of biochar, were more likely to capture OH^−^, undergo deprotonation and carry negative charges. As illustrated in Equations (14) and (15), the transformation of surface -COOH and -OH functional groups to their deprotonated forms (-COO⁻ and -O⁻) resulted in enhanced surface electronegativity of HBS50-K0.5M, consequently improving its affinity for Pb^2+^ adsorption [23].

-OH + OH^−^ → -O^−^ + H_2_O (14)

-COOH + OH^−^ → -COO^−^ + H_2_O (15)

The influence of sorbent dosage on Pb^2+^ removal was examined using HBS50-K0.5M quantities varying between 0.01 and 0.04 g. Figure 5b demonstrates a progressive enhancement in Pb^2+^ removal efficiency corresponding to increased sorbent dosage across this range. The growth trend stabilized when the adsorbent dosage exceeded 0.03 g. However, the sorbent capacity decreased significantly with the increase in adsorbent dose, which can be attributed to the oversaturation of adsorption sites caused by excessive sorbent dose [22,24]. Furthermore, excessive biochar application may induce competitive deprotonation among functional groups, resulting in reduced surface electronegativity per unit mass compared to lower doses. This phenomenon could potentially diminish the Pb^2+^ adsorption capacity due to site dilution effects [10,12].

The influence of sorption time, as well as the initial concentration of Pb^2+^ on the Pb^2+^ sorption behavior of HBS50-K0.5M, is shown in Figure 5c,d. The initial efficiency of Pb^2+^, which subsequently experienced a deceleration in growth rate, culminated in the approach towards equilibrium values (50.76% and 345.8 mg/g) around 120 min of sorption time—significantly outpacing previous benchmarks [8,25]. It was observed that HBS50-K0.5M, while significantly enhancing the equilibrium sorption capacity, still retained the rapid equilibrium attainment characteristic of the unmodified hydrothermal biochar (HBS50). Despite physicochemical analyses indicating that ultrasonic-assisted KOH activation caused some disruption to the pore structure, it concurrently reduced the particle size and improved dispersion. This enhancement in dispersion increased the possibility of interactions between the biochar’s active sites and Pb^2+^ ions, thereby leading to an enhanced sorption rate [12,14]. As shown in Figure 5d, at higher Pb^2+^ concentrations (100 mg/L), the minimum dose (0.8 g/L) of HBS50-K0.5M can achieve a removal efficiency of over 99.3%, manifesting its excellent Pb^2+^ removal ability. Although the removal efficiency gradually diminished with increasing initial Pb^2+^ concentrations, the sorption capacity concurrently increased [12,14]. At lower initial Pb^2+^ concentrations, the increment in biochar’s Pb^2+^ sorption capacity was pronounced as the initial concentration increased. When the initial concentration attained around 300 mg/L, the sorption capacity reached 334.0 mg/g, after the growth rate of sorption capacity tended to be slow and gradual. This slowing was attributed to higher Pb^2+^ content in the solution, elevating the chances of interactions between active sorption sites and Pb^2+^ compared with lower concentration solutions [12,14,19]. Therefore, the initial increase in sorption capacity was notable, but as the capacity grew to a certain level, implying that a large number of the biochar’s active sites were nearing saturation, the trend in capacity growth decelerated [14,26].

To comprehend the kinetics and mechanisms behind the sorption of Pb^2+^ by HBS50-K0.5M, a number of kinetic equations were applied in order to fit the data obtained from the sorption experiments. The kinetic equations included the PF and PS equations. As illustrated in Figure 5e, within 40 min, the rate of increase in Pb^2+^ sorption capacity was notably rapid. After 40 min, the rate of increase slowed down. When the time reached around 120 min, the amplification in sorption capacity became gentle and approached the equilibrium value (345.8 mg/g). According to Table 2, the fitting regression coefficient (R2) for the PS kinetic equation was greater than that for the PF. Therefore, it can be inferred that the PS kinetic model more closely approximates the sorption behavior of HBS50-K0.5M toward Pb^2+^. This manifested that the sorption rate in this process was predominantly driven by chemical interactions (such as complexation, precipitation, ion exchange, etc.) between the sorbent and the sorbate [27].

In addition, the Langmuir and Freundlich isotherms were employed to model the data obtained from sorption experiments. The results are illustrated in Figure 5f and Table 2. Initial observations from the graph indicated a sharp rise in sorption capacity with increasing amounts of Pb^2+^, which then transitions to a more gradual increase, eventually reaching an equilibrium capacity of approximately 345.8 mg/g. The graph shows that the Langmuir isotherm model is more closely aligned with the experimental data. Furthermore, as indicated by Table 2, the Langmuir model’s correlation coefficient (R2) exceeded that of the Freundlich model, revealing a better fit for the sorption characteristics, thereby implying that the sorption of Pb^2+^ by HBS50-K0.5M occurred via monolayer sorption [27]. Moreover, according to the Langmuir model’s fit, the theoretical maximum Pb^2+^ sorption capacity (*qm*) of HBS50-K0.5M was predicted to be 353.3 mg/g. Table 3 compares and shows the Pb^2+^ adsorption capacities of different hydrothermal biochars reported in the previous literature. It has been observed that ultrasonic-assisted KOH-activated hydrothermal biochar for removing Pb^2+^ from the water has some advantages.

#### 3.2.3. Effect of Coexisting Ions

**Figure 6:**
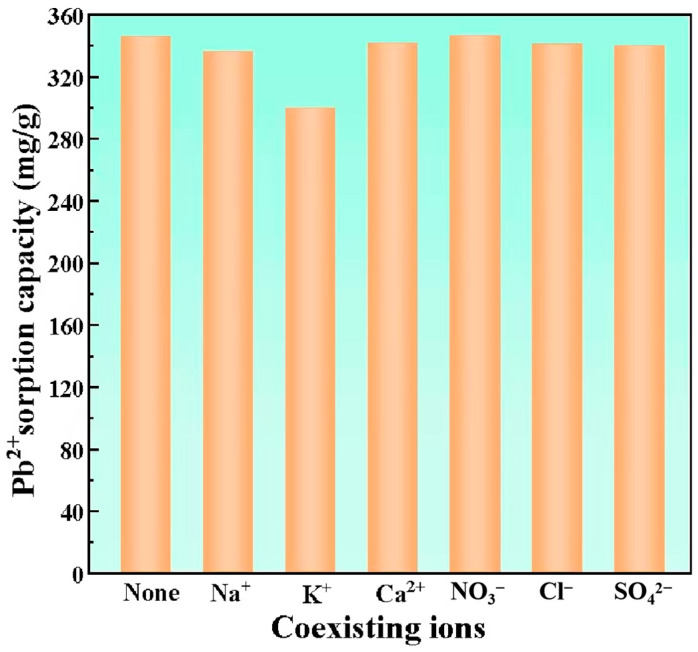
Coexisting ion factors. Starting Pb^2+^ concentration = 120 mg/L, starting pH = 5, temperature = 25 °C, duration = 120 min, volume of solution = 25 mL, concentration of various coexisting ions in the solution = 0.1 mol/L, and adsorbent = 20 mg were the experimental conditions.

Industrial wastewater matrices typically contain diverse competing ions that may occupy available adsorption sites, thereby reducing Pb^2+^ uptake through competitive sorption mechanisms [31]. To study this phenomenon, a range of coexisting anions and cations were used and tested. These included Na^+^, K^+^, Ca^2+^, NO^3−^, Cl^−^, and SO4^2−^. The test subjects were HBS50-K0.5M. The findings are depicted in Figure 6. It was self-evident that various coexisting anions in the solution do not significantly impact the Pb^2+^ sorption capacity of HBS50-K0.5M. This was attributable to the electrostatic repulsion between the surface of HBS50-K0.5M, with its negative charge, and the anions in the solution, thus rendering the influence of anions on Pb^2+^ minuscule. Considering the various coexisting cations, the influence of Na^+^ and Ca^2+^ on the Pb^2+^ sorption capacity of HBS50-K0.5M was minimal. Nevertheless, the presence of K+ in the solution caused a noticeable reduction in the Pb^2+^ sorption capacity of HBS50-K0.5M, dropping from 345.8 mg/g to 299.8 mg/g. This indicated that ion exchange between K^+^ and Pb^2+^ was one of the mechanisms through which HBS50-K0.5M adsorbed Pb^2+^. In solutions with high concentrations of K^+^, the release of biochar into the solution was inhibited, which weakens the ion exchange and leads to a decrease in adsorption capacity [32].

### 3.3. Sorption Mechanisms

**Figure 7:**
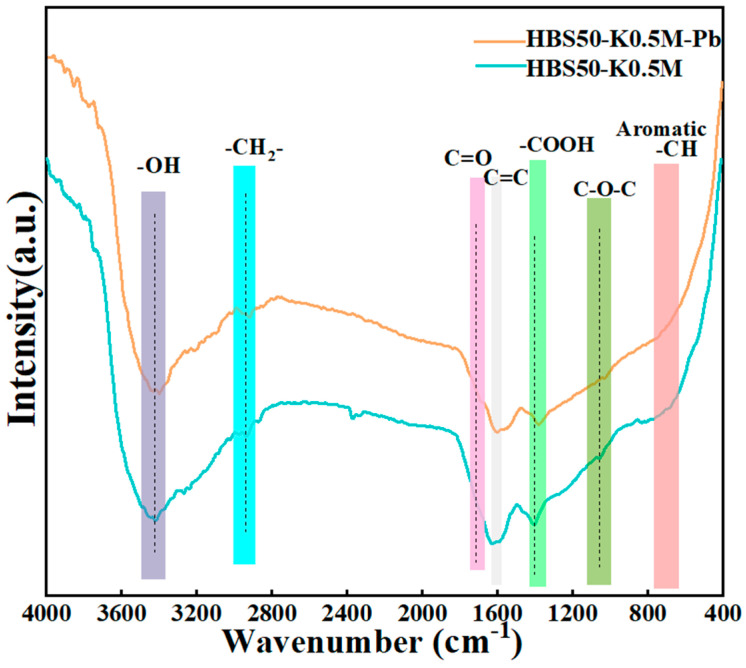
FTIR spectra of HBS50−K0.5M and HBS50-K0.5M−Pb.

The changes in functional groups were found with FT-IR after Pb^2+^ sorption by the ultrasonic-assisted KOH activation biochars, as shown in Figure 7. It was clear that the binding of Pb atoms to the surface of the biochar caused the overall sorption intensity to decrease following sorption. Moreover, the distinctive peaks of -OH (3450 cm^−1^) as well as -COOH (1390 and 1220 cm^−1^) moved to the right, possibly due to complexation between Pb atoms as well as functional groups that incorporate oxygen [8]. The broadening and shifting of peaks at C=C (1590 cm^−1^) as well as C=O (1680 cm^−1^) was observed, suggesting π-π interactions between HBS50-K0.5M with Pb^2+^ [8].

**Figure 8:**
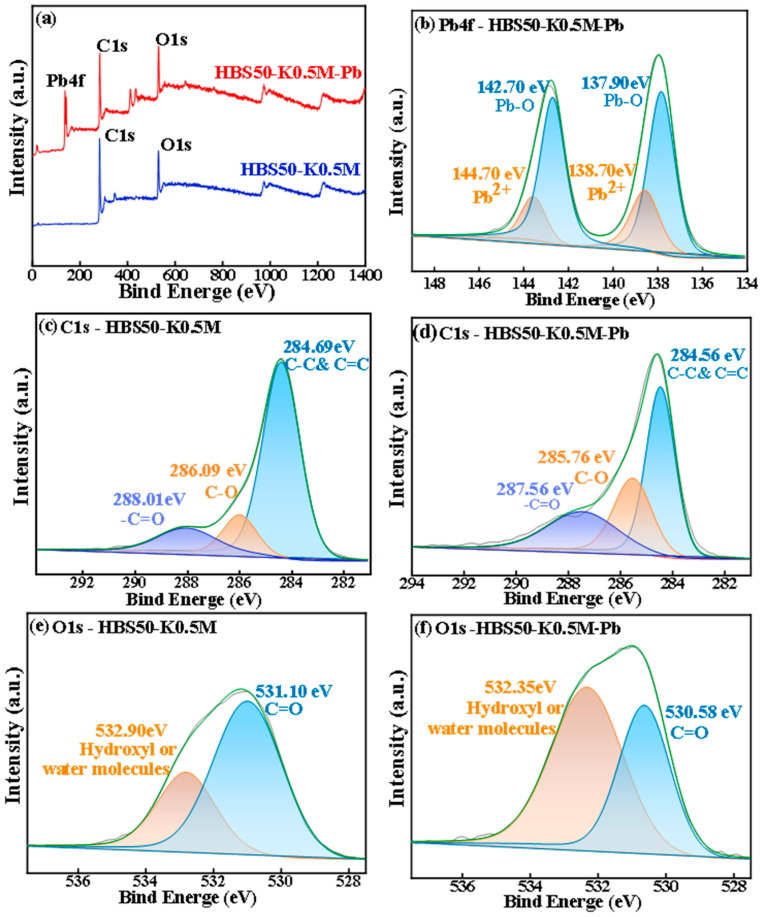
XPS spectra of HBS50-K0.5M before and after the reaction of (**a**) total photoelectron energy spectrum, (**b**) Pb4f after the reaction, (**c**) C1s before the reaction, (**d**) C1s after the reaction, (**e**) O1s before the reaction, (**f**) O1s after the reaction.

XPS analysis of HBS50-K0.5M and HBS50-K0.5M-Pb was carried out, and the pre-adsorption XPS revealed that the adsorbed material consisted of the elements C and O, while the post-adsorption consisted of the elements C, O, and Pb, as shown in Figure 8. In Figure 8a, the HBS50-K0.5M-Pb spectrum exhibits characteristic peaks of Pb 4f, indicating the successful binding of Pb atoms to the biochar surface. Figure 8b shows the Pb 4f spectrum of HBS50-K0.5M-Pb, where most Pb atoms on the biochar surface were found in Pb-O, as well as a small amount of Pb^2+^, indicating that most Pb atoms underwent complex reactions with oxygen-containing functional groups resulting in the generation of complex compounds, such as 2O-Pb, 2COO-Pb, COO-Pb-O. Furthermore, a tiny percentage of Pb atoms are bonded to the surface of the biochar by π-π bonds, as well as electrostatic attraction [16]. The presence of chelating interactions between Pb atoms and oxygen-containing functional groups is further demonstrated by the C 1s spectra shown in Figure 8c,d, which show a small decrease in the binding energies of -C=O (288.01 eV), as well as C-O (286.09 eV) bonds after sorption [33,34]. The π-π bonding effect on unsaturated carbon bonds was thought to be responsible for the shift seen at 284.56 eV, which corresponds to C-C as well as C=C bonds. According to the O1s spectra in Figure 8e,f, the peak at 531.10 eV denotes C=O functionality, while the peak at 532.90 eV represents hydroxyl groups or water molecules [8]. The slight decrease in binding energies for these peaks after sorption implies a chelating interaction between Pb atoms and oxygen-containing functional groups [35].

**Figure 9:**
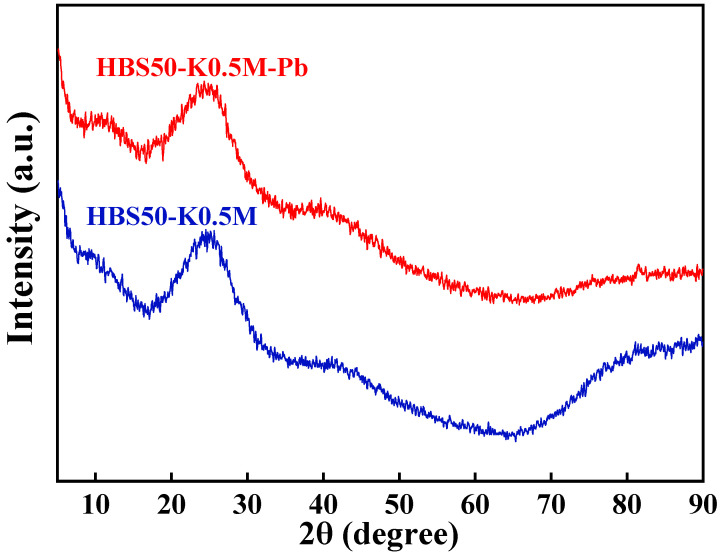
XRD spectra of HBS50-K0.5M before and after sorption.

According to previous studies, ion exchange and precipitation reactions have been considered potential mechanisms for biochar’s sorption of Pb^2+^ [36]. To investigate these two potential sorption mechanisms, this study utilized ICP–OES to measure the concentrations of Na, Mg, K, and Ca, i.e., common alkali metal elements, in the solution before and after sorption (Table 4) and conducted X-ray diffraction (XRD) analyses on samples HBS50-K0.5M and HBS50-K0.5M-Pb to verify the presence of any Pb(II) precipitates (as shown in Figure 9). In the XRD pattern of HBS50-K0.5M and HBS50-K0.5M-Pb, a broad peak near 23° associated with the (002) diffraction plane of disordered amorphous carbon was observed [37,38]. Furthermore, no precipitates containing Pb elements were detected, indicating that precipitation was not the dominant mechanism in the sorption process of HBS50-K0.5M for Pb^2+^. Table 4 indicates that the concentrations of Na, Mg, as well as Ca in the sorption solution, remained almost unchanged before and after the sorption experiment, while the concentration of K element in the solution increased by 20.9 mg/L after sorption, indicating that K ions attached to the surface of biochar by complexation or electrostatic attraction and exchange with Pb^2+^ ions in solution take part in adsorption. The exchange of Pb^2+^ ions in solution is shown in Equation (16).

(Biochar)-2K + Pb^2+^ → (Biochar)- Pb + 2K^+^ (16)

**Figure 10:**
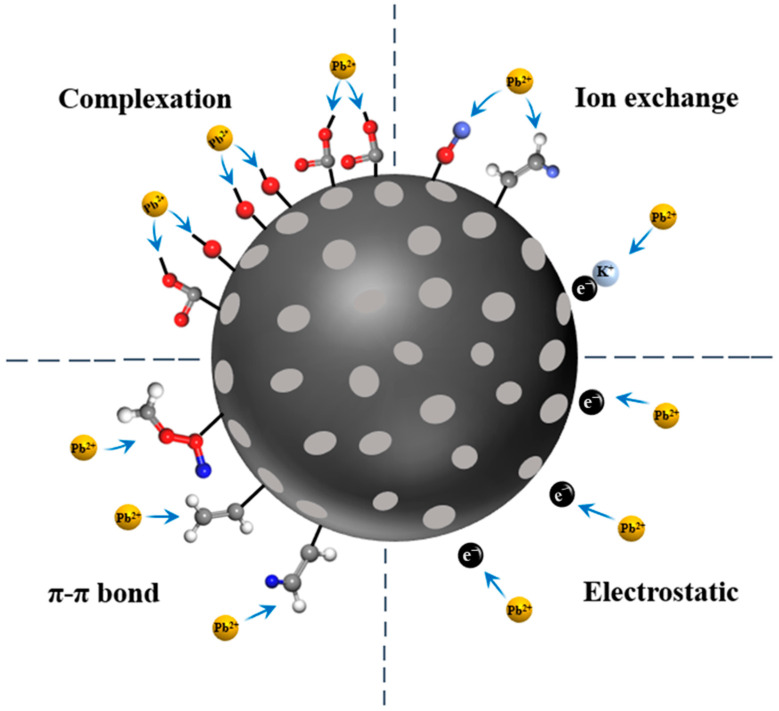
Mechanisms of Pb^2+^ adsorption by HBS50-K0.5M.

Based on the conclusions drawn from sorption experiments and the aforementioned characterizations, the mechanism of KOH ultrasonic modified hydrothermal biochar (HBS50-K0.5M) for Pb^2+^ sorption was proposed as follows: During the sorption process, the -OH and -COOH groups on the biochar surface deprotonated in water, becoming negatively charged (as shown in Equation (14) as well as Equation (15)), and attracted Pb^2+^ to the biochar surface through electrostatic interactions [12]. Then, Pb^2+^ ions attached to the biochar surface through four sorption mechanisms: complexation with oxygen-containing functional groups (as shown in Equations (16)–(19), ion exchange with K atoms attached to the biochar surface (as shown in Equation (16)), π-π electron donor–acceptor interactions, and electrostatic attraction. A schematic diagram of the sorption mechanism is presented in Figure 10.

2-O^−^ + Pb^2+^ → 2O-Pb^+^ (17)

2-COO^−^ + Pb^2+^ → 2COO-Pb (18)

-O^−^ + -COO^−^ + Pb^2+^ → COO-Pb-O (19)

## 4. Conclusions

This study investigated the adsorption of Pb^2+^ by ultrasonic-assisted KOH-modified hydrothermal biochar, revealing the intrinsic mechanism of activation. The results showed that ultrasonic-assisted KOH activation has a remarkable effect on the physicochemical properties of hydrothermal biochar. Compared with HBS50, the etching effect induced by KOH activation contributes to a decrease in particle size, as well as an increase in the number of O-containing functional groups on the biochar surface, which can provide more available adsorption sites for Pb^2+^ adsorption. The hydrothermal biochar HBS50-K0.5M modified with 0.5 mol/L KOH solution exhibits the best sorption capacity with a maximum sorption capacity of 345.8 mg/g, which is significantly better than HBS50 (195.9 mg/g). This modified hydrothermal biochar is environmentally friendly and is expected to be an efficient adsorbent for Pb^2+^ removal.

## Figures and Tables

**Table 1:** The elemental compositions and the corresponding elemental ratios, zero potential (pHpzc), preparation yield, specific surface area and pore structure parameters of IHSC, HBS50 and KOH ultrasonic modified biochars.

Sample	Elemental Content (%)				O/C	Specific Surface Area (m^2^/g)	Total Pore Volume (cm^3^/g)	Average Pore Diameter (nm)	pH_pzc_
	C	O	H	N					
IHSC	46.58	45.10	1.77	1.17	0.968	----	----	----	----
HBS50	56.53	34.30	2.54	0.25	0.607	359.11	0.357	8.95	3.35
HBS50-K0.25M	52.98	37.59	2.52	0.28	0.710	30.71	0.122	19.15	4.61
HBS50-K0.5M	51.48	38.66	2.64	0.21	0.751	21.33	0.099	18.81	4.49
HBS50-K1M	57.18	33.20	2.69	0.31	0.581	17.61	0.082	18.65	4.33

**Table 2:** Fitted parameters of the kinetic and isotherm sorption models for Pb^2+^ onto HBS50-K0.5M.

Sorption kinetics	Pseudo-first-order Model			Pseudo-second-order Model		
	*k*_1_ (min^−1^)	*q_e_* (mg/g)	*R* ^2^	*k*_2_ (min^−1^)	*qe* (mg/g)	*R* ^2^
	0.102	284.5	0.867	7.073	344.7	0.988
Sorption isotherm	Langmuir isotherm			Freundlich isotherm		
	*k_l_* (min^−1^)	*q_m_* (mg/g)	*R* ^2^	*k_f_* (min^−1^)	*n*	*R* ^2^
	3.123	353.3	0.963	146.66	0.155	0.891

**Table 3:** Comparison of preparation yield and Pb^2+^ sorption capacity of hydrothermal biochars at the temperature of 25℃.

Hydrothermal Biochars	Preparation Yield (%)	pH	Q_m_ (mg/g)	Ref.
Rice husk hydrothermal biochars	----	5.0	1.84	[17]
MnFe_2_O_4_-activated sludge hydrothermal biochar	----	Natural pH	174.2	[25]
H_3_PO_4_-activated banana peel hydrothermal biochar	28.9	Natural pH	356.2	[10]
K_2_CO_3_-activated Laminaria japonica hydrothermal biochar	16.6	Natural pH	108.0	[28]
MnFe_2_O_4_-activated Undaria pinnatifida roots hydrothermal biochar	----	5.0	175.4	[29]
Urea/ZnCl_2_-activated Camellia sinensis waste hydrothermal biochar	43.7	Natural pH	73.1	[30]
H_2_SO_4_-activated industrial hemp stalk core-driven hydrothermal biochar	49.2	5.0	195.9	[8]
KOH-modified industrial hemp straw core hydrothermal biochar	39.0	5.0	345.8	This work

**Table 4:** Contents of alkali metal elements in solution before and after the reaction.

Solutions	Alkali Metal Elemental Content (mg/L)			
	Na	Mg	K	Ca
Before	0.389	0.478	0	0.620
After	0.421	0.422	20.90	0.700

## Data Availability

The original contributions presented in this study are included in the article. Further inquiries can be directed to the corresponding authors.
